# Electromyography–Force Relation and Muscle Fiber Conduction Velocity Affected by Spinal Cord Injury

**DOI:** 10.3390/bioengineering10020217

**Published:** 2023-02-06

**Authors:** Le Li, Huijing Hu, Bo Yao, Chengjun Huang, Zhiyuan Lu, Cliff S. Klein, Ping Zhou

**Affiliations:** 1 Institute of Medical Research, Northwestern Polytechnical University, Xi’an 710072, China; 2 Institute of Biomedical Engineering, Chinese Academy of Medical Sciences & Peking Medical College, Beijing 100006, China; 3 Department of Neuroscience, Baylor College of Medicine, Houston, TX 77030, USA; 4 School of Rehabilitation Science and Engineering, University of Health and Rehabilitation Sciences, Qingdao 266072, China; 5 Rehabilitation Research Institute, Guangdong Work Injury Rehabilitation Center, Guangzhou 510440, China

**Keywords:** spinal cord injury (SCI), muscle fiber conduction velocity (MFCV), surface electromyography (EMG), EMG–force relation

## Abstract

A surface electromyography (EMG) analysis was performed in this study to examine central neural and peripheral muscle changes after a spinal cord injury (SCI). A linear electrode array was used to record surface EMG signals from the biceps brachii (BB) in 15 SCI subjects and 14 matched healthy control subjects as they performed elbow flexor isometric contractions from 10% to 80% maximum voluntary contraction. Muscle fiber conduction velocity (MFCV) and BB EMG–force relation were examined. MFCV was found to be significantly slower in the SCI group than the control group, evident at all force levels. The BB EMG–force relation was well fit by quadratic functions in both groups. All healthy control EMG–force relations were best fit with positive quadratic coefficients. In contrast, the EMG–force relation in eight SCI subjects was best fit with negative quadratic coefficients, suggesting impaired EMG modulation at high forces. The alterations in MFCV and EMG–force relation after SCI suggest complex neuromuscular changes after SCI, including alterations in central neural drive and muscle properties.

## 1. Introduction

Spinal cord injuries (SCIs) can cause motor dysfunction including loss of maximal strength and impaired force control that is partially explained by altered muscle activation [1]. In patients with incomplete SCIs, there is a reduction in nervous system activation of skeletal muscle below the lesion. Hence, some motor units (motor neurons) of a muscle may not be recruited despite maximal effort, due to denervation or loss of central neural activation, whereas others may discharge at lower than normal rates [2,3]. Recording of muscle activity by electromyography (EMG) has proved to be useful for evaluating central and peripheral determinants of motor dysfunction [4,5]. In contrast to clinical measures of motor function, EMG is sensitive enough to detect muscle activity after SCI in the absence of palpable muscle contraction and joint movement [6]. Abnormal EMG findings from impaired muscles after SCI include long-lasting involuntary motor activation [7,8], loss of functioning motor units [9,10,11,12,13,14,15], impaired motor unit voluntary control [16,17,18], and muscle fiber denervation and reinnervation [3,19,20]. EMG has demonstrated to be a valuable tool for assessment of paralyzed muscle changes in persons with SCI.

The relation between surface EMG amplitude and voluntary isometric muscle force has been explored in people with motor disorders such as stroke [21,22,23,24]. Alterations in EMG–force relation compared with matched healthy control subjects have been observed, that may be related to altered motor control and motor unit properties. In contrast with stroke, few have examined the EMG–force relation after SCI. Thomas and colleagues reported linear (or curvilinear) EMG–force relations in the triceps brachii of persons with chronic SCI that were similar to healthy controls [25].

The EMG–force relation has mainly been examined with conventional single channel surface electrodes. High density surface EMG (HD-sEMG) arrays provide advantages over conventional single channel EMG [26]. For example, Jordanic et al. compared the performance of HD-sEMG and single channel EMG in the upper limb of SCI subjects and found that spatial activation of motor units was dependent on the contraction intensities and the type of exercise, and the related spatial features can improve the identification of specific co-activation patterns during motor performance [27]. Among various HD-sEMG array designs, a one-dimensional linear electrode array is convenient to use [28]. The linear electrode array can simultaneously measure EMG from different locations of the muscle fibers and thus may detect activity in severely paralyzed muscles that may be undetected using conventional single channel EMG. Linear electrode arrays have other useful applications including estimation of locations of innervation zones (IZ) and muscle fiber conduction velocity (MFCV) [29,30,31,32].

In this study, we completed an analysis of surface EMG from linear electrode array attached on the biceps brachii (BB) muscle in persons with SCI and a matched group of healthy controls. The purpose of the study was to characterize the BB MFCV and EMG–force relationship, and whether they are affected by SCI.

## 2. Materials and Methods

### 2.1. Subjects

The participants of this study included 15 SCI survivors (3 female and 12 male, 44.6 ± 16.1 years,) with injury duration 1–36 years, injury level from C2–C8 and American Spinal Injury Association (ASIA) impairment scale A to D. More information on injury level and ASIA impairment scale can be found in [33]. All SCI survivors were recruited from the outpatient clinic of TIRR Memorial Hermann Hospital (Houston, TX, USA). Their clinical characteristics are summarized in Table 1. In addition, 14 able-bodied subjects (3 female and 11 male, 39.7 ± 12.4 years) with no known history of neuromuscular disorder were recruited as the control group. There was no age difference between the two groups (*p* = 0.37). This study was approved by the Institutional Review Board of the University of Texas Health Science Center at Houston and TIRR Memorial Hermann Hospital and performed in accordance with the Declaration of Helsinki. All subjects gave written consents (or had a witnessed verbal consent if unable to write) before participating in the experiment.

### 2.2. Experiment

**Figure 1:**
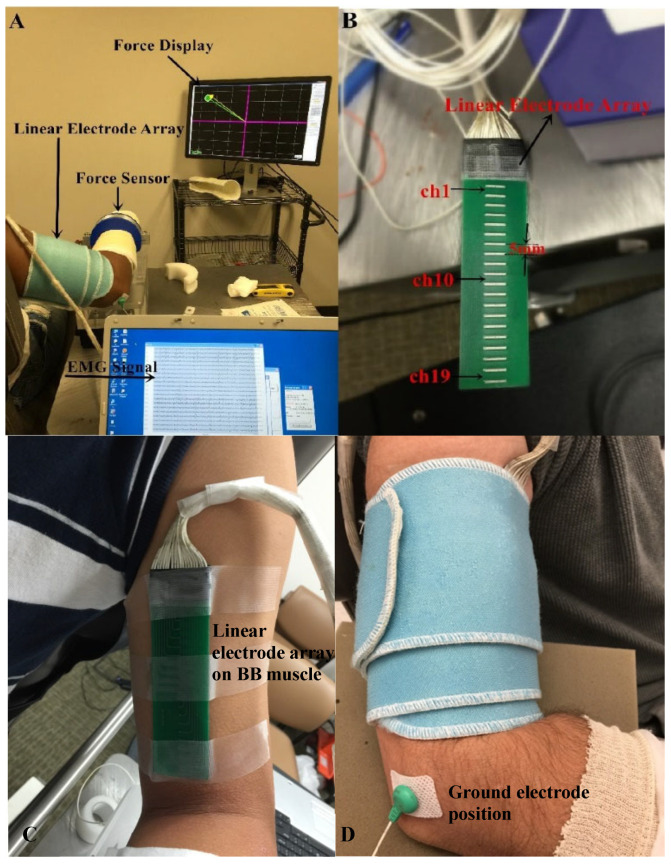
Experimental setup. (**A**) Force display and EMG recording; (**B**) The linear electrode array used for surface EMG recording; (**C**) The placement of the linear electrode array on BB muscle belly; (**D**) The fixation of the linear electrode array.

The participants were seated in a chair which is adjustable for comfortable height and were instructed to hold the arm position with the elbow in 90° of flexion and the shoulder in 45° of abduction during the data collection (Figure 1A). A Velcro strap was used to restrain the shoulder and trunk from moving during the experiment. The wrist and forearm were immobilized in a handmade fiberglass cast and placed on a fixed platform. The wrist joint was restricted inside a ring interface, which was mounted to the platform. The ring interface was connected to a load cell (ATI, Apex, NC, USA). Force signals were recorded with a sampling frequency of 2 kHz and digitized by a BNC-2090A data acquisition board (National Instruments, Austin, TX, USA). The fiberglass cast helped to fix the upper limb well and minimize the movement and variation between subjects. 

The maximum voluntary contraction (MVC) of the weaker side of the SCI subjects (determined by self-report and clinical assessment) and the non-dominant side of the control subjects was determined. Each subject conducted three MVC trials of the BB muscle. The largest one was defined as the MVC value. The target force of 10–80% MVC (in 10% MVC increment) was marked as a circle with a line connecting the circle and the center of the computer screen (representing the rest state) (Figure 1A). For each desired force level, there was a computer-generated cursor tracking the force in real time. The subject was asked to move the cursor to follow the force line. When the target circle was reached (indicated by color change in the cursor), the subject was instructed to keep the cursor as stable as possible inside the target circle. In the case of having difficulty in reaching the target (especially for the high force level tasks), the subject was verbally encouraged to control the cursor as close as possible to the target. The whole process (moving the cursor to the target and holding the cursor) lasted for at least 10 s. Each subject was allowed to perform practice trials to become familiar with the contraction task before data recording. The sequence of different muscle contraction levels was randomized. Each muscle contraction level was repeated twice. The subjects were explicitly instructed not to change or move trunk position during task performance. To avoid mental or muscle fatigue, subjects were allowed to have at least 2 min break between trials. 

Surface EMG was captured from the BB muscle by a linear electrode array designed and manufactured in our lab. The array has 20 silver bars, with each bar being 10 mm in length and 1 mm in width. The inter-bar distance is 5 mm (Figure 1B). Skin preparation was performed with sandpaper, alcohol pads, and conductive gel. The array was positioned over the midline of the BB muscle longitudinally from the bicipital groove to the biceps tendon insertion (Figure 1C). Such placement ensured that the electrode array covered the major portion of the muscle. In addition, self-adhesive cuff was used to wrap the linear electrode array and secure a good attachment on the skin surface of the BB muscle during the experiment (Figure 1D). The reference electrode was attached on the lateral condyle of the subject’s tested arm. Surface EMG signals were recorded via the Porti EMG acquisition system (TMS International, Oldenzaal, The Netherlands). The sampling frequency was 2 kHz per channel. There is a 1st order low pass filter before the ADC with a −3 dB point at 4.8 kHz. The ADC of the Porti has a digital sinc3 filter with a cutoff frequency of 0.27× sample frequency. 

### 2.3. Data Analysis

#### 2.3.1. Data Preprocessing

Surface EMG and force data were processed offline in MATLAB (MathWorks, Natick, MA, USA). A 6th order Butterworth (10–500 Hz) was applied to the EMG signals. The power line interference in the EMG signal was eliminated using a spectrum interpolation algorithm [34]. Force signals were manually inspected to select a relatively stable 5 s segment. Surface EMG and force signals within the epochs were extracted for further analysis.

#### 2.3.2. Calculation of MFCV

Prior to the analysis of MFCV, EMG signals were differentiated between consecutive channels to generate 19 channels of bipolar signals. The IZ was determined by either visual inspection or analysis of the bipolar signals. The IZ was estimated to be the channel with the lowest amplitude, or between the channels that demonstrated reverse signal polarity and a clear pattern of bidirectional signal propagation from the IZ channel to the tendons [35]. The MFCV was determined based on detection of the temporal delay between adjacent single differential channels. Specifically, MFCV was defined and computed as *d*/*τ*, where *d* is the inter-electrode distance between the channels and *τ* is the time delay between two channels (calculated from cross-correlation analysis). Channels containing IZ or adjacent to IZ were excluded for MFCV estimation. The MFCV calculated at each contraction level was averaged over all contraction levels for further comparison between groups.

#### 2.3.3. EMG–Force Relation

The force signal from individual trials was averaged over the selected epoch. The corresponding surface EMG amplitude was obtained by calculation of the root mean square (RMS) value from each channel. Channels close to the proximal and distal tendons were excluded from analysis. The channel producing the maximal RMS values (by evaluating the eight target force contractions and the MVC) was used for estimation of the EMG–force relation. Next, the RMS values from the selected channel were averaged across the 2 trials for each force level. The force was also averaged across the trials. The RMS and force values were then normalized to the MVC values for determination of the EMG–force relation.

The EMG–force relation was estimated in each SCI and control subject. Given that curvilinear EMG–force relation has been widely reported for large muscles such as BB [36,37], we applied quadratic fitting to describe the BB EMG–force relation. The quadratic equation was expressed as: *y* = a*x*^2^ + b*x* + c, where *x* represents force and *y* represents EMG amplitude. The coefficient of determination (R^2^) for quadratic fitting was calculated for each subject.

### 2.4. Statistical Analysis

Descriptive statistics were performed. A normal distribution of MVC and MFCV values was confirmed by the Kolmogorov–Smirnov test. The independent t test was applied to compare the difference of MVC between the SCI and healthy control groups. A linear-mixed effects model was applied to analyze the main effects of group (SCI and Control), force (8 levels from 10% to 80% MVC) and the interaction of the two main effects on MFCV. The coefficient of determination of the quadratic fitting of the EMG–force relation was calculated for both control and SCI groups. Statistical analysis was conducted using SPSS (SPSS Inc., Chicago, IL, USA) with a significance level of *p* < 0.05. All values in the text are presented as mean ± SD.

## 3. Results

**Figure 2:**
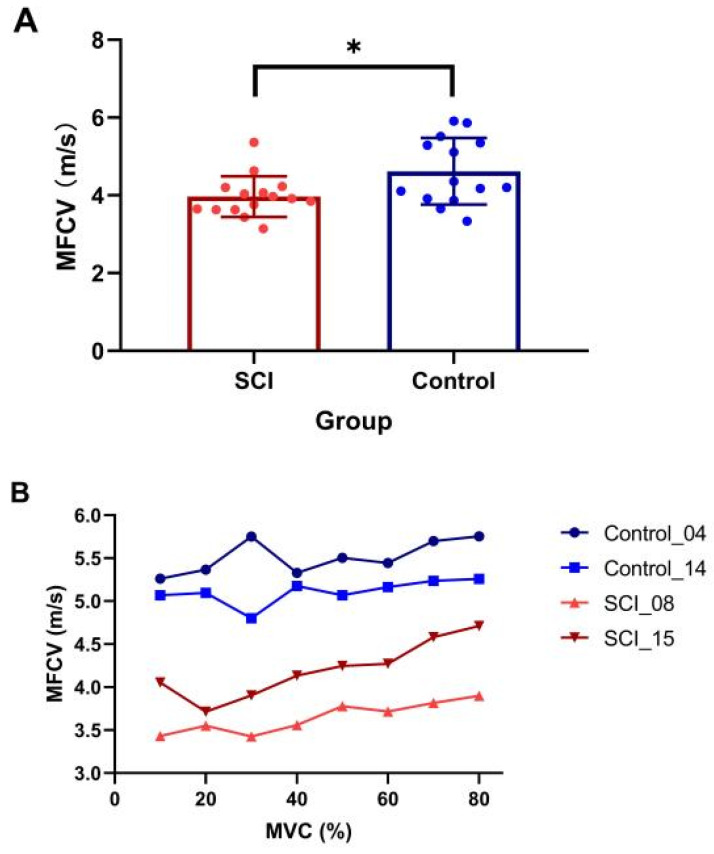
Comparison of MFCV between SCI and control subjects. (**A**) Mean and individual MFCVs in each group (* *p* < 0.05, error bar represents standard deviation); (**B**) MFCV at the different target forces in two subjects from SCI and control groups, respectively.

The SCI participants were significantly weaker compared to the controls (SCI MVC: 98.5 ± 69.9 N, range: 11.9–307.6 N; control MVC: 212.7 ± 111.3 N, range: 70.6–314.9 N, *p* = 0.005). Examination of differences of average MFCV value revealed a significantly slower value in the SCI group compared with the healthy control group (SCI: 3.97 ± 0.55 m/s, control: 4.62 ± 0.86 m/s, *p* = 0.025, Figure 2A). The results showed a significant main effect of group (presence of SCI) (*β* = −0.87, SE = 0.29, t = −2.97, *p* = 0.005), while the main effect of contraction level (*β* = −0.04, SE = 0.02, t = −1.67, *p* = 0.096) and interaction of the two main effects (*β* = 0.002, SE = −0.004, t = 1.79, *p* = 0.075) were not significant. Although a trend of increasing MFCV with muscle contraction level was observed in some subjects (Figure 2B), linear-mixed effects model analysis indicated that MFCV was not significantly related to the different target forces.

**Figure 3:**
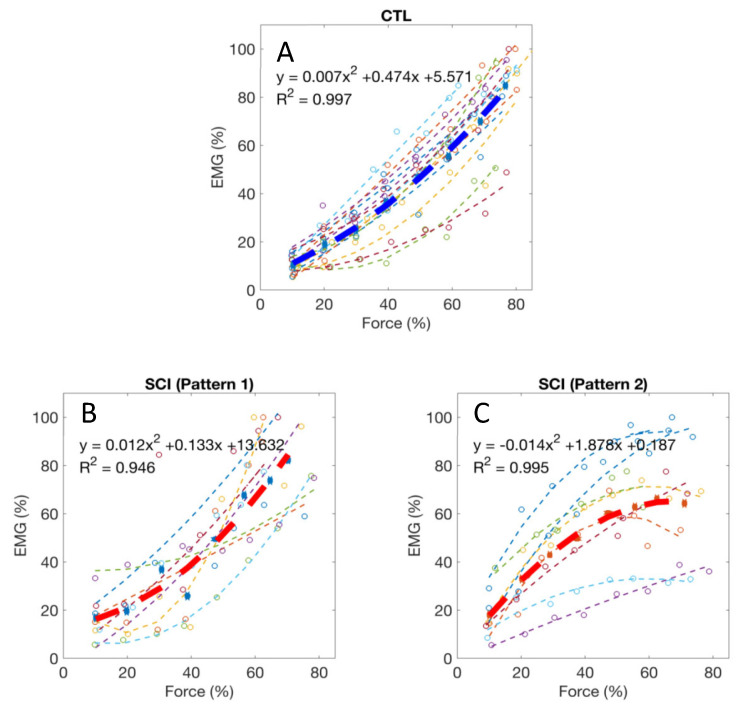
(**A**) Normalized EMG–force relations from all healthy control subjects and the averaged relation (the thick line); (**B**,**C**) Two typical patterns of EMG–force relations from all the tested SCI subjects and the averaged relations (the thick lines).

Normalized EMG–force relations in all the tested healthy control subjects and the averaged relation are shown in Figure 3A. For all 14 healthy control subjects, the EMG–force relation was well fit by a quadratic function (R^2^ = 0.96, range: 0.89–0.99). All the control subjects had a positive quadratic coefficient (i.e., a > 0), suggesting that EMG tended to increase relatively more than force during the stronger target contractions. In contrast, a more diverse EMG–force relation was observed in the SCI subjects, although data for the group was also well fit by a quadratic function (R^2^ = 0.87, range: 0.50–0.99). Two different quadratic patterns were observed after SCI. Among the 15 tested SCI subjects, seven had a positive quadratic coefficient (i.e., a > 0, Figure 3B), consistent with the responses in the controls. The other eight SCI subjects had a negative quadratic coefficient (i.e., a < 0, Figure 3C), suggesting that EMG tended to increase relatively less than force during the stronger target contractions. The two SCI sub-groups with negative and positive quadratic coefficients did not have significant differences in age, years post injury, ASIA scale, neurological level, and MVC force (*p* > 0.05).

## 4. Discussion

A linear electrode array was applied in this study to examine the BB partially paralyzed by cervical SCI. The average MVC of the examined muscles was approximately 50% of healthy control subjects, and this weakness likely reflects both central neural (i.e., paralysis) and peripheral muscular changes. In the subacute or chronic stage after SCI, muscles innervated by spinal segments at and caudal to the SCI are prone to atrophy from both denervation and disuse [38]. A decrease in the number of motor units or axons following spinal motor neuron death could occur [9,10,11,12,13,14,15]. There were only a few motor units that remained under voluntary control since the injury interrupted many of the descending inputs to the motor neuron pool [39]. The disturbances of motor neuron control and contractile properties persist in chronic SCI survivors, and represent an important source of muscular weakness and increased fatigability [40]. These neurophysiological changes were also reflected in the current linear electrode array EMG analysis of the SCI subjects, focused on the MFCV and the EMG–force relation.

MFCV, which is directly related to membrane excitability, can reflect the dynamic changes and redistribution of ions during voluntary muscle contraction [41]. MFCVs calculated in the BB muscles were reported to range from 3.4 ± 0.2 to 5.0 ± 0.6 m/s in healthy subjects [28,42]. Our results from healthy control subjects (4.62 ± 0.86 m/s) are in similar range with the previous findings. Changes in MFCV have been reported after neuromuscular disorders. For example, BB MFCV during isometric contractions were found to be significantly slower in patients with Duchenne muscular dystrophy compared to healthy controls [43]. MFCV was also shown to be significantly slower in paretic muscles of stroke survivors [29]. In this study, BB MFCV was significantly slower after SCI compared to control subjects. This could be due to muscle fiber atrophy or degeneration of large motor units after SCI. Given that the BB has motor unit recruitment range up to 80% MVC, a clear correlation was expected between muscle force and MFCV to be revealed in current study. However, our results indicated no significant correlation between the averaged MFCV and muscle contraction level for both groups. For the SCI group, this is likely due to complex neuromuscular changes that may compromise the relationship between MFCV and muscle force. Admittedly, our results from healthy control subjects are somewhat different from most of the previous literatures [44,45,46], although a similar finding was also reported in a recent study that BB MFCV of healthy control subjects increased only slightly but non-significantly with force [47]. According to size principle, later recruited motor units are supposed to have larger muscle fiber diameters and thus higher MFCV. Although some of the healthy control subjects showed an increase trend of MFCV with muscle contraction level, group analysis did not reveal a significant relation. There might be multiple factors that likely compromise the MFCV of the healthy control subjects in this study, which were also suggested in previous studies. For example, Masuda et al. (1996) [48] examined MFCVs from vastus lateralis, tabialis anterior, and BB muscles of seven healthy subjects. Although increased MFCV was observed with increasing force of the vastus lateralis muscle, the results from BB muscle showed that the MFCV reduced rapidly with time before the muscle contraction force reached the designed target levels of 70% or 90% MVC. MFCV at these larger force levels was smaller than that at 50% MVC and then consequently MFCV in the BB showed no dependent on the contraction levels. These results suggest that although MFCV basically increases with muscle contraction force but this relation can become unclear when MFCV decreases rapidly with time. Other factors may also contribute to compromising the relation such as variability in interference surface EMG, variability between different sessions (especially at higher contraction force), muscle temperature variability (which may also affect MFCV) [49], and muscle fatigue. Although muscle fatigue was a controlled factor during experiment and subjects were allowed sufficient rest, it would be difficult to completely avoid its effect on MFCV, especially at high force levels when large and fast-fatigable motor units are recruited [50]. 

The EMG–force relation was also examined in this study, which can provide additional insights pertaining to neuromuscular changes in pathological conditions. Application of a linear electrode array can characterize the EMG–force relation unconfounded by muscle IZ effects on the EMG signal. This is important because surface EMG parameters can be significantly affected by IZs, and the uncertainty of electrode locations (with respect to the IZ) might compromise the signal interpretation [51,52]. Both linear and nonlinear EMG–force relations were reported in the literature [36,37]. For small muscles such as the first dorsal interosseous (FDI) whose force generation is dominated by motor unit rate coding, a linear EMG–force relation is often observed. For large muscles such as BB, motor unit recruitment takes an important role in muscle force generation, and the progressively recruited motor units have larger action potentials, increase EMG more than force despite the effect of action potential amplitude cancellation, thus resulting in a nonlinear EMG–force relation. In this study, we observed that for all healthy control subjects, the quadratic term coefficient of the EMG–force relation fitting was positive, suggesting that EMG increased faster than force, which is consistent with previous reports [36].

An interesting finding is that for the SCI group, diverse EMG–force relations were observed. In about half of the SCI subjects, a negative quadratic term coefficient of the EMG–force relation fitting was revealed, indicating that EMG tended to increase slower than force. There are various factors that may contribute to this EMG–force relation change after SCI. Previously, the effects of different motor unit property changes on the EMG–force relation were systematically investigated by simulating activities of motor neuron pool, surface EMG and force of the FDI muscle [53]. For example, it was found that reductions in motor unit firing rate would tend to increase the slope of EMG–force relation, which was experimentally confirmed in stroke subjects [21,22,23]. Jahanmiri-Nezhad et al. found a trend of decreased slope of the EMG–force relation in the FDI muscle of patients with amyotrophic lateral sclerosis compared with healthy control subjects, which could be related to selective degeneration of motor units with high threshold or a change in motor unit contractile properties [54]. In the current study of the SCI BB, the unusual negative quadratic term coefficients could be caused by motor unit property changes after SCI, such as the loss of large motor units, and altered motor unit recruitment as well as firing behavior. For example, Johanson et al. found two of the four SCI subjects had significantly reduced motor neuron recruitment and high firing rates, likely a compensatory effect of dramatic motor neuron loss after SCI, while the other two subjects with stronger elbow extension had relatively normal recruitment and firing rates [55]. It is worth noting that there are various interactive factors that can influence the EMG–force relation in different ways. Those positive quadratic term coefficients of the fitting in SCI subjects consistent with the healthy control group might be viewed as a collective effect of various factors, which can drive the EMG–force relation in opposite directions.

There are several limitations in the present study. We solely applied global surface EMG parameters and it might be difficult to differentiate or quantify various motor unit properties that may contribute to the changes in surface EMG. Surface EMG decomposition is required to perform analysis at the motor unit level. Given that it is more ideal to perform surface EMG decomposition using 2-dimensional electrode arrays which provide EMG recordings not only parallel to but also perpendicular to muscle fibers, surface EMG decomposition was not attempted in this study. Motor unit number, size, and control property changes after SCI can readily be examined through 2-dimensional high density surface EMG recording and decomposition in future studies [56,57]. Considering that surface electrode only records superficial regions of a muscle, intramuscular recording with needle or fine wire electrodes is necessary in order to capture activity of deeper motor units in the muscle. As computational modeling provides a useful approach in neuromuscular performance investigations [53,58], a delicate simulation analysis incorporating experimental motor unit behaviors can help understand the global surface EMG parameter alterations after SCI. The current study focused on MFCV and the relation of EMG amplitude and muscle force for a relatively steady segment of signals, while there are more advanced or complex signal processing methods which can be applied in data analysis. For example, wavelet transform is promising to explore time and frequency dependence of the examined parameters [59]. In this study, EMG was not recorded from synergistic and antagonistic muscles, although a previous SCI study found that BB coactivation did not have a major effect on the triceps brachii EMG–force relation [25]. Simultaneous recording from synergistic and antagonistic muscles is suggested in the future study, which can assess the potential effects of the “sharing load” strategy, especially during strong contractions. In addition, the shoulder and trunk position may influence EMG signal measurement [60], and this should be considered in data analysis and interpretation. Finally, this study is limited by a relatively small subject number for performing meaningful sub-group analysis.

In summary, this study presents findings from a linear electrode array surface EMG examination of the BB in chronic cervical SCI subjects. The results demonstrated significantly slower MFCV in SCI subjects compared with healthy controls. The EMG–force relation was also altered in a subset of the SCI participants. Using quadratic fitting of the EMG–force relation, approximately half of the SCI participants demonstrated a negative quadratic term coefficient, possibly reflecting impaired motor unit control at high forces. In contrast, positive quadratic coefficients were observed for all healthy control subjects. These findings suggest both central neural and peripheral muscular changes in the BB after SCI. 

## Figures and Tables

**Table 1:** SCI subject information.

Subject No	Age (years)	Gender	Years Past Injury	Neurological Level	ASIA Impairment Scale
1	38	Female	10	C6	B
2	47	Male	10	C5	C
3	50	Male	26	C5	D
4	23	Male	9	C3	A
5	39	Male	3	C6	D
6	62	Female	11	C8	D
7	65	Male	2	C2	C
8	32	Male	1	C8	C
9	59	Male	8	C5	D
10	50	Female	30	C5	C
11	25	Male	2.5	C4	D
12	54	Male	36	C4	C
13	19	Male	4	C2	D
14	36	Male	4	C5	C
15	71	Male	3	C4	C

## Data Availability

The data that support the findings of this study are available from the corresponding author upon reasonable request.

## References

[1] Sherwood A.M., McKay W.B., Dimitrijević M.R. (1996). Motor control after spinal cord injury: Assessment using surface EMG. Muscle Nerve.

[2] Thomas C.K., Zaidner E.Y., Calancie B., Broton J.G., Bigland-Ritchie B.R. (1997). Muscle weakness, paralysis, and atrophy after human cervical spinal cord injury. Exp. Neurol..

[3] Thomas C.K., Bakels R., Klein C.S., Zijdewind I. (2014). Human spinal cord injury: Motor unit properties and behavior. Acta Physiol..

[4] Calancie B., Molano M.R., Broton J.G., Bean J.A., Alexeeva N. (2001). Relationship between EMG and muscle force after spinal cord injury. J. Spinal Cord Med..

[5] de Vargas Ferreira V.M., Varoto R., Cacho Ê.W.A., Cliquet A. (2012). Relationship between function, strength and electromyography of upper extremities of persons with tetraplegia. Spinal Cord.

[6] Calancie B., Molano M.D.R., Broton J.G. (2000). Neural plasticity as revealed by the natural progression of movement expression-both voluntary and involuntary—In humans after spinal cord injury. Prog. Brain Res..

[7] McKay W.B., Ovechkin A.V., Vitaz T.W., de Paleville D.G.T., Harkema S.J. (2011). Long-lasting involuntary motor activity after spinal cord injury. Spinal Cord.

[8] Zijdewind I., Thomas C.K. (2001). Spontaneous motor unit behavior in human thenar muscles after spinal cord injury. Muscle Nerve.

[9] Xiong G.X., Zhang J.W., Hong Y., Guan Y., Guan H. (2008). Motor unit number estimation of the tibialis anterior muscle in spinal cord injury. Spinal Cord.

[10] Li X., Jahanmiri-Nezhad F., Rymer W.Z., Zhou P. (2012). An examination of the motor unit number index (MUNIX) in muscles paralyzed by spinal cord injury. IEEE Trans. Inf. Technol. Biomed..

[11] Li L., Li X., Liu J., Zhou P. (2015). Alterations in multidimensional motor unit number index of hand muscles after incomplete cervical spinal cord injury. Front. Hum. Neurosci..

[12] Zong Y., Lu Z., Chen M., Li X., Stampas A., Deng L., Zhou P. (2021). CMAP scan examination of the first dorsal interosseous muscle after spinal cord injury. IEEE Trans. Neural Syst. Rehabil. Eng..

[13] Lu Z., Chen M., Zong Y., Li X., Zhou P. (2022). A Novel Analysis of CMAP Scans from Perspective of Information Theory: CMAP Distribution Index (CDIX). IEEE Trans. Biomed. Eng..

[14] Li J., Zhu Y., Li Y., He S., Wang D. (2020). Motor unit number index detects the effectiveness of surgical treatment in improving distal motor neuron loss in patients with incomplete cervical spinal cord injury. BMC Musculoskelet. Disord..

[15] Witt A., Fuglsang-Frederiksen A., Finnerup N.B., Kasch H., Tankisi H. (2020). Detecting peripheral motor nervous system involvement in chronic spinal cord injury using two novel methods: MScanFit MUNE and muscle velocity recovery cycles. Clin. Neurophysiol..

[16] Smith H.C., Davey N.J., Savic G., Maskill D.W., Ellaway P.H., Frankel H.L. (1999). Motor unit discharge characteristics during voluntary contraction in patients with incomplete spinal cord injury. Exp. Physiol..

[17] Zijdewind I., Thomas C.K. (2003). Motor unit firing during and after voluntary contractions of human thenar muscles weakened by spinal cord injury. J. Neurophysiol..

[18] Thomas C.K., Broton J.G., Calancie B. (1997). Motor unit forces and recruitment patterns after cervical spinal cord injury. Muscle Nerve.

[19] Riley D.A., Burns A.S., Carrion-Jones M., Dillingham T.R. (2011). Electrophysiological dysfunction in the peripheral nervous system following spinal cord injury. PM&R.

[20] Zhang X., Li X., Tang X., Chen X., Chen X., Zhou P. (2020). Spatial filtering for enhanced high-density surface electromyographic examination of neuromuscular changes and its application to spinal cord injury. J. Neuroeng. Rehabil..

[21] Zhou P., Li X., Rymer W.Z. (2013). EMG-force relations during isometric contractions of the first dorsal interosseous muscle after stroke. Top. Stroke Rehabil..

[22] Suresh N.L., Concepcion N.S., Madoff J., Rymer W.Z. (2015). Anomalous EMG-force relations during low-force isometric tasks in hemiparetic stroke survivors. Exp. Brain Res..

[23] Bhadane M., Liu J., Rymer W.Z., Zhou P., Li S. (2016). Re-evaluation of EMG-torque relation in chronic stroke using linear electrode array EMG recordings. Sci. Rep..

[24] Zhang X., Wang D., Yu Z., Chen X., Li S., Zhou P. (2017). EMG-torque relation in chronic stroke: A novel EMG complexity representation with a linear electrode array. IEEE J. Biomed. Health Inform..

[25] Thomas C.K., Tucker M.E., Bigland-Ritchie B. (1998). Voluntary muscle weakness and co-activation after chronic cervical spinal cord injury. J. Neurotrauma.

[26] Drost G., Stegeman D.F., van Engelen B.G., Zwarts M.J. (2006). Clinical applications of high-density surface EMG: A systematic review. J. Electromyogr. Kinesiol..

[27] Jordanic M., Rojas-Martínez M., Mañanas M.A., Alonso J.F. (2016). Spatial distribution of HD-EMG improves identification of task and force in patients with incomplete spinal cord injury. J. Neuroeng. Rehabil..

[28] Merletti R., Farina D., Gazzoni M. (2003). The linear electrode array: A useful tool with many applications. J. Electromyogr. Kinesiol..

[29] Yao B., Zhang X., Li S., Li X., Chen X., Klein C.S., Zhou P. (2015). Analysis of linear electrode array EMG for assessment of hemiparetic biceps brachii muscles. Front. Hum. Neurosci..

[30] Conrad M.O., Qiu D., Hoffmann G., Zhou P., Kamper D.G. (2017). Analysis of muscle fiber conduction velocity during finger flexion and extension after stroke. Top. Stroke Rehabil..

[31] Jahanmiri-Nezhad F., Li X., Barkhaus P.E., Rymer W.Z., Zhou P. (2014). A clinically applicable approach for detecting spontaneous action potential spikes in amyotrophic lateral sclerosis with a linear electrode array. J. Clin. Neurophysiol..

[32] Li X., Lu Z., Wang I., Li L., Stampas A., Zhou P. (2021). Assessing redistribution of muscle innervation zones after spinal cord injuries. J. Electromyogr. Kinesiol..

[33] Burns S., Biering-Sørensen F., Donovan W., Graves D., Jha A., Johansen M., Jones L., Krassioukov A., Kirshblum S., Mulcahey M.J. (2012). International Standards for Neurological Classification of Spinal Cord Injury, Revised 2011. Top. Spinal Cord Inj. Rehabil..

[34] Mewett D.T., Reynolds K.J., Nazeran H. (2004). Reducing power line interference in digitised electromyogram recordings by spectrum interpolation. Med. Biol. Eng. Comput..

[35] Beck T.W., Housh T.J., Cramer J.T., Mielke M., Hendrix R. (2008). The influence of electrode shift over the innervation zone and normalization on the electromyographic amplitude and mean power frequency versus isometric torque relationships for the vastus medialis muscle. J. Neurosci. Methods.

[36] Woods J.J., Bigland-Ritchie B. (1983). Linear and non-linear surface EMG/force relationships in human muscles. An anatomical/functional argument for the existence of both. Am. J. Phys. Med..

[37] Zhou P., Rymer W.Z. (2004). Factors governing the form of the relation between muscle force and the EMG: A simulation study. J. Neurophysiol..

[38] Biering-Sørensen B., Kristensen I.B., Kjaer M., Biering-Sørensen F. (2009). Muscle after spinal cord injury. Muscle Nerve.

[39] Thomas C.K., del Valle A. (2001). The role of motor unit rate modulation versus recruitment in repeated submaximal voluntary contractions performed by control and spinal cord injured subjects. J. Electromyogr. Kinesiol..

[40] Klein C.S., Häger-Ross C.K., Thomas C.K. (2006). Fatigue properties of human thenar motor units paralysed by chronic spinal cord injury. J. Physiol..

[41] McGill K.C., Lateva Z.C. (2011). History dependence of human muscle-fiber conduction velocity during voluntary isometric contractions. J. Appl. Physiol..

[42] Nishihara K., Chiba Y., Moriyama H., Hosoda M., Suzuki Y., Gomi T. (2009). Noninvasive estimation of muscle fiber conduction velocity distribution using an electromyographic processing technique. Med. Sci. Monit..

[43] Martinez A.C., Terradas J.M.L. (1990). Conduction velocity along muscle fibers in situ in Duchenne muscular dystrophy. Arch. Phys. Med. Rehabil..

[44] Rainoldi A., Galardi G., Maderna L., Comi G., Lo Conte L., Merletti R. (1999). Repeatability of surface EMG variables during voluntary isometric contractions of the biceps brachii muscle. J. Electromyogr. Kinesiol..

[45] Sadoyama T., Masuda T. (1987). Changes of the average muscle fiber conduction velocity during a varying force contraction. Electroencephalogr. Clin. Neurophysiol..

[46] Andreassen S., Arendt-Nielsen L. (1987). Muscle fibre conduction velocity in motor units of the human anterior tibial muscle: A new size principle parameter. J. Physiol..

[47] Klaver-Krol E.G., Hermens H.J., Vermeulen R.C., Klaver M.M., Luyten H., Henriquez N.R., Zwarts M.J. (2021). Chronic fatigue syndrome: Abnormally fast muscle fiber conduction in the membranes of motor units at low static force load. Clin. Neurophysiol..

[48] Masuda T., Sadoyama T., Shiraishi M. (1996). Dependence of average muscle fiber conduction velocity on voluntary contraction force. J. Electromyogr. Kinesiol..

[49] Troni W., DeMattei M., Contegiacomo V. (1997). The effect of temperature on conduction velocity in human muscle fibers. J. Electromyogr. Kinesiol..

[50] Arendt-Nielsen L., Mills K.R., Forster A. (1989). Changes in muscle fiber conduction velocity, mean power frequency, and mean EMG voltage during prolonged submaximal contractions. Muscle Nerve.

[51] Beck T.W., Housh T.J., Cramer J.T., Weir J.P. (2007). The effect of the estimated innervation zone on EMG amplitude and center frequency. Med. Sci. Sport. Exerc..

[52] Huang C., Klein C.S., Meng Z., Zhang Y., Li S., Zhou P. (2019). Innervation zone distribution of the biceps brachii muscle examined using voluntary and electrically-evoked high-density surface EMG. J. Neuroeng. Rehabil..

[53] Zhou P., Suresh N.L., Rymer W.Z. (2007). Model based sensitivity analysis of EMG-force relation with respect to motor unit properties: Applications to muscle paresis in stroke. Ann. Biomed. Eng..

[54] Jahanmiri-Nezhad F., Hu X., Suresh N.L., Rymer W.Z., Zhou P. (2014). EMG-force relation in the first dorsal interosseous muscle of patients with amyotrophic lateral sclerosis. NeuroRehabilitation.

[55] Johanson M.E., Lateva Z.C., Jaramillo J., Kiratli B.J., McGill K.C. (2013). Triceps Brachii in incomplete tetraplegia: EMG and dynamometer evaluation of residual motor resources and capacity for strengthening. Top. Spinal Cord Inj. Rehabil..

[56] Chen M., Zhou P. (2016). A Novel Framework Based on FastICA for High Density Surface EMG Decomposition. IEEE Trans. Neural Syst. Rehabil. Eng..

[57] Chen M., Zhang X., Chen X., Zhou P. (2018). Automatic Implementation of Progressive FastICA Peel-Off for High Density Surface EMG Decomposition. IEEE Trans. Neural Syst. Rehabil. Eng..

[58] Sybilski K., Mazurkiewicz L., Jurkokc J., Michnik R., Malachowski J. (2021). Evaluation of the effect of muscle forces implementation on the behavior of a dummy during a head-on collision. Acta Bioeng. Biomech..

[59] von Tscharner V., Barandun M. (2010). Wavelet based correlation and coherence analysis reveals frequency dependent motor unit conduction velocity of the abductor pollicis brevis muscle. J. Electromyogr. Kinesiol..

[60] Dejneka A., Malachowski J., Mazurkiewicz L. (2022). Identification of muscle movements and activity by experimental methods for selected cases—Stage. Acta Bioeng. Biomech..

